# Supplementary material for: RIF1 promotes replication fork protection and efficient restart to maintain genome stability
Source: Nat Commun. 2019 Jul 23;10:3287. doi: 10.1038/s41467-019-11246-1 (PMC6650494; doi:10.1038/s41467-019-11246-1)
Supplement: Supplementary file 1 — Supplementary Information [file 41467_2019_11246_MOESM1_ESM.pdf]

Supplementary information

RIF1 promotes replication fork protection and efficient restart to maintain genome stability

Mukherjee et al.

*Correspondence and request of materials should be addressed to:
ARC (a.raychaudhuri@erasmusmc.nl)

Supplementary Figures

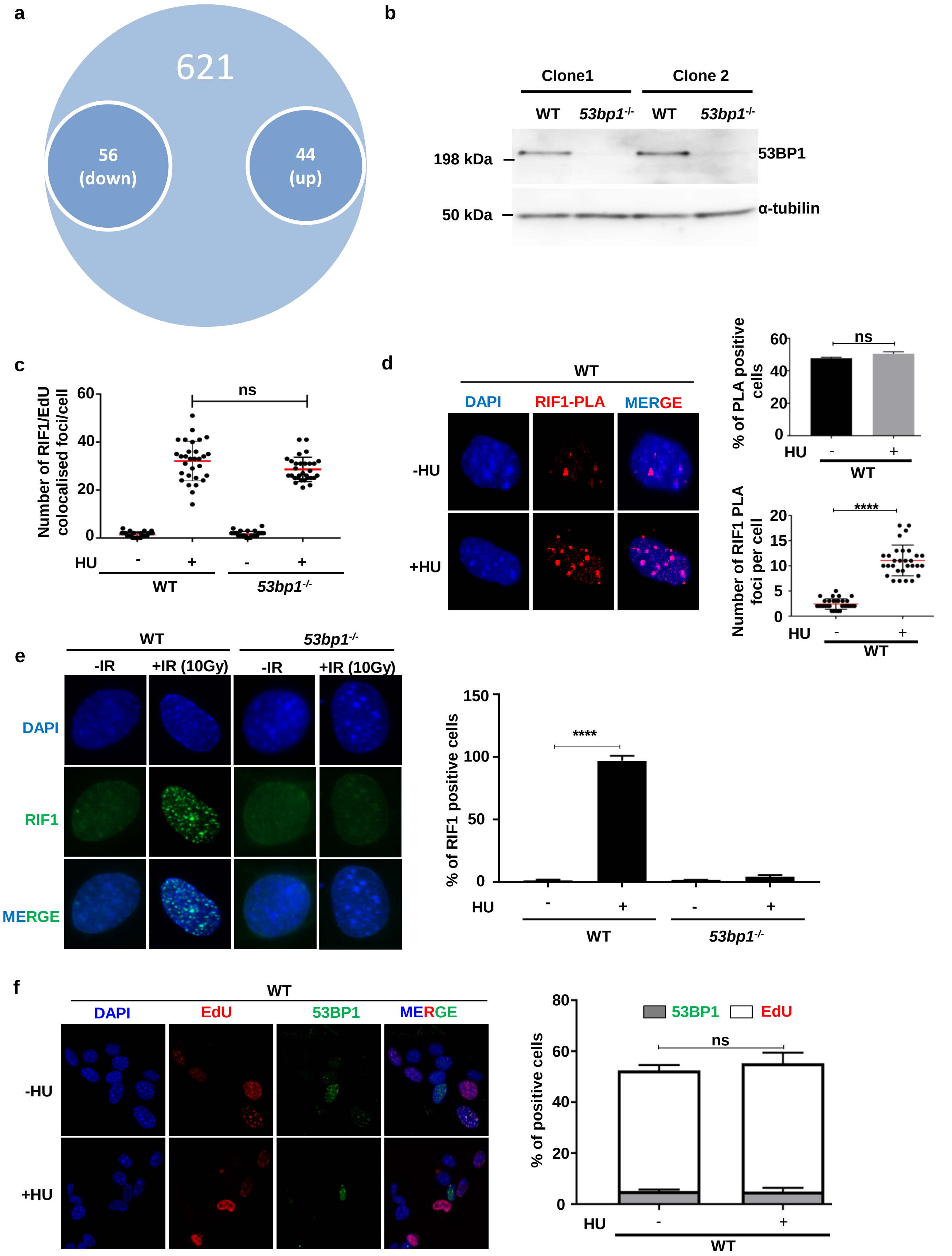

Supplementary Figure.1. Identification of novel factors enriched at stalled replication forks . a) Venn- Diagram representing the number of proteins which are enriched and decreased at stalled replication forks in the iPOND coupled to mass spectrometry data. **b)** Western blot analysis for checking the protein levels from two different clones of WT and *53bp1*$^{-/-}$ MEFs. Lysates were prepared for WT and *53bp1*$^{-/-}$ MEFs and western blots were carried out against 53BP1 antibody. Tubulin was taken as loading control. **c)** Quantitation of the co-localization of RIF1 with EdU per cell with in presence or absence of HU (unpaired t-test, n.s., non-significant) obtained from three independent experiments. **d)** RIF1 is recruited at replication fork sites upon replication stress. Representative micrographs from proximity ligation assay (PLA) showing the co-localization of RIF1 and EdU to sites of DNA replication in presence or absence of HU treatment in WT cells. Nucleus was stained with DAPI. Adjoining graph on top panel quantifies the percentage of PLA positive cells (unpaired t-test, ns non-significant, error bars represent standard deviation). 104 cells were quantified for each experiment, bottom panel quantitation of the number of the RIF1 PLA foci per cell **e)** WT and 53bp1$^{-/-}$ MEFs were irradiated with or without 10 Gy and allowed to recover for 2h. RIF1 (green) foci formation was assessed.The nucleus is stained with DAPI (blue). Adjoining graph quantifies the percentage of RIF1 positive foci. (Unpaired t-test, p-value < 0.0001; error bars represent standard deviations). 105 cells were quantified for each experimental set. **f)** 53BP1 foci formation in presence and absence of HU treatment. MEF WT cells were treated with 20 µM EdU for 15 min, followed by HU treatment for 3h (4mM). Cells were fixed, permeabilized and subsequently stained for 53BP1. Click-it reaction was carried out for visualization of EdU. Adjoining graph shows the quantification of co-localization of 53BP1 and EdU before and after treatment. (Unpaired t-test, ns non-significant, error bars represent standard deviation).

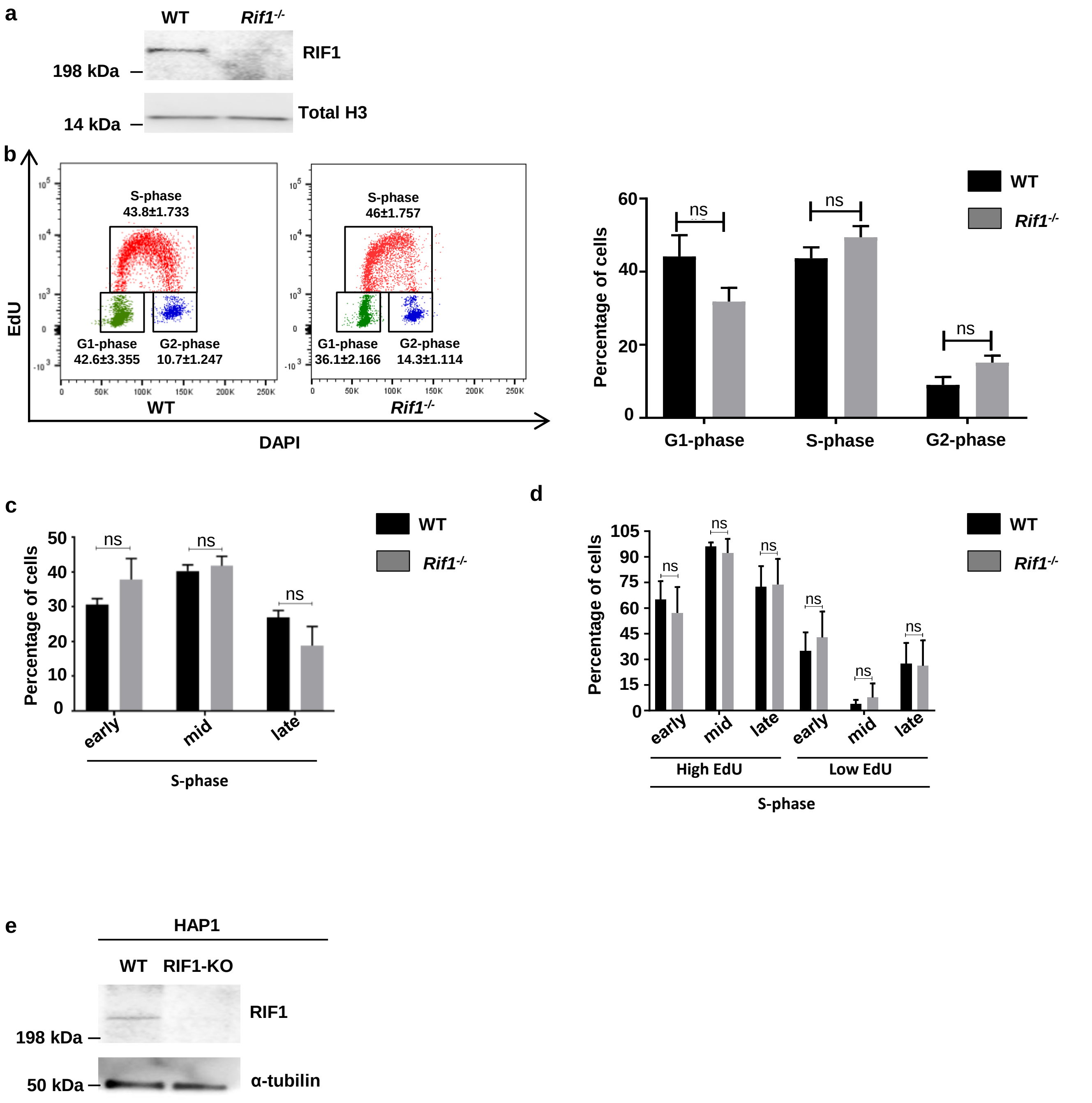

Supplementary Figure.2. Comparison of S-phase progression in WT and *Rif1*$^{-/-}$ cells. a) RIF1 protein levels in WT and Rif1$^{-/-}$ MEFs**.** Cell lysates from WT and *Rif1*$^{-/-}$ MEFs were probed with antibody against mRIF1 and tubulin was taken as loading control **b)** FACS analysis of DNA synthesis (by EdU incorporation) was tested. EdU-negative cells represent G1 and G2/M cells. Quantification of the percentage of cells in each phase of cell cycle was done from three independent experiments. **c)** Percentage of sub S-phase cells were obtained for WT and *Rif1*$^{-/-}$ MEFs from three independent experiments. **d)** Intensity of EdU in each sub S-phase was assessed and unpaired t-test was done for all the three analysis **b-c-d** (ns, not significant). **e)** Cell lysates from WT and RIF1-KO HAP1 cells were probed with antibody against hRIF1 to check the expression of RIF1. Tubulin is used as loading control.

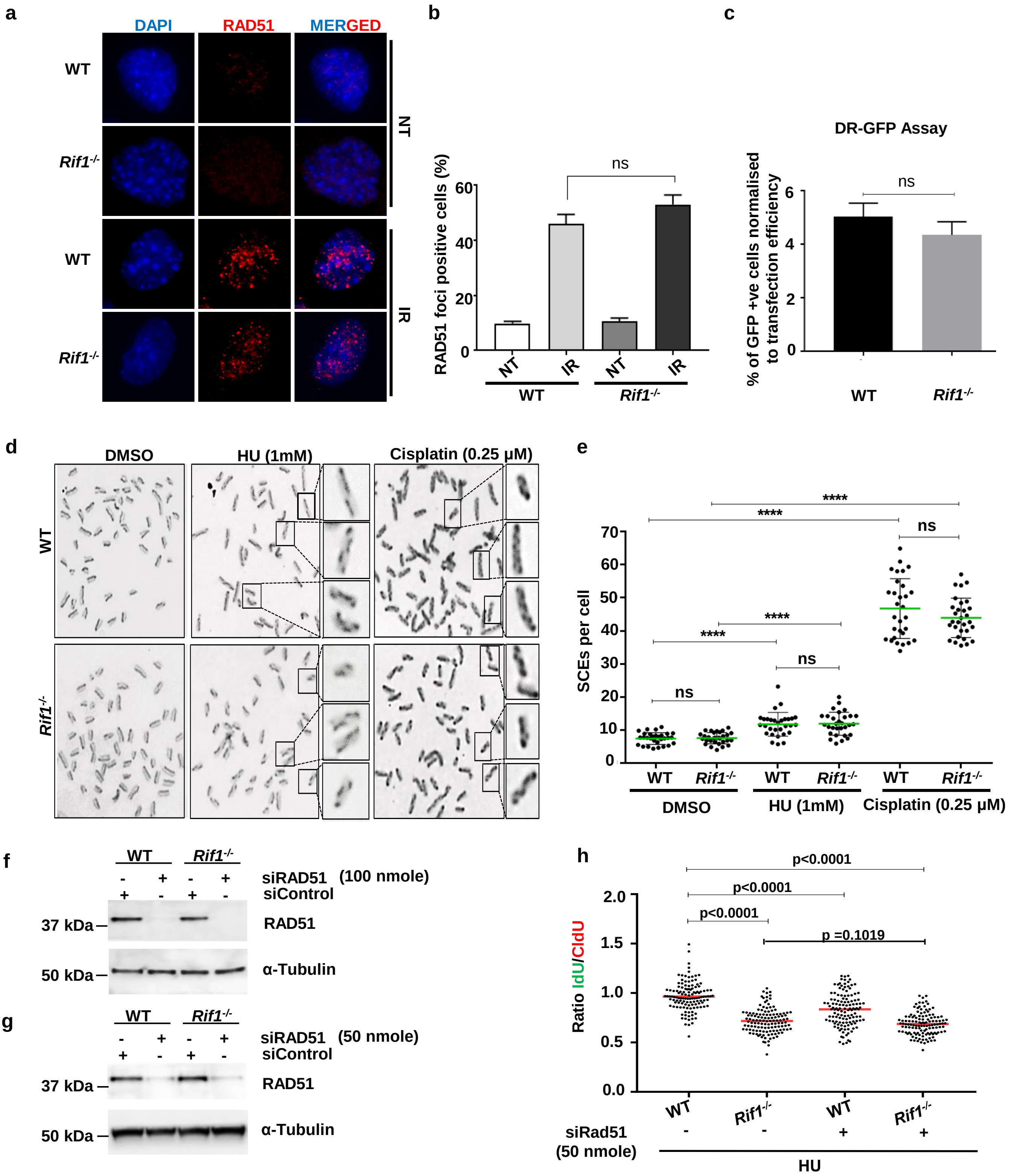

a
DAPI
RAD51
MERGED
WT
Rif1-/-
WT
Rif1-/-
NT
IR
b
RAD51 foci positive cells (%)
ns
0
20
40
60
NT
IR
NT
IR
WT
Rif1-/-
c
DR-GFP Assay
% of GFP +ve cells normalised to transfection efficiency
ns
0
2
4
6
WT
Rif1-/-
d
DMSO
HU (1mM)
Cisplatin (0.25 μM)
WT
Rif1-/-
e
SCEs per cell
0
10
20
30
40
50
60
70
****
****
ns
****
****
ns
ns
WT
Rif1-/-
WT
Rif1-/-
WT
Rif1-/-
DMSO
HU (1mM)
Cisplatin (0.25 μM)
f
WT
Rif1-/-
- + - +
+ - + -
siRAD51 (100 nmole)
siControl
37 kDa
RAD51
50 kDa
α-Tubulin
g
WT
Rif1-/-
- + - +
+ - + -
siRAD51 (50 nmole)
siControl
37 kDa
RAD51
50 kDa
α-Tubulin
h
Ratio IdU/CldU
0.0
0.5
1.0
1.5
2.0
p<0.0001
p<0.0001
p<0.0001
p =0.1019
WT
Rif1-/-
WT
Rif1-/-
siRad51 (50 nmole)
- - + +
HU

Supplementary Figure.3. Comparison of HR-proficiency between WT and Rif1$^{-/-}$ MEFs. **a)** Representative micrographs showing the co-localization of RAD51 in (red) at the sites of DSBs in WT and *Rif1*$^{-/-}$ MEFs before and after irradiation (IR). Nucleus was stained with DAPI (blue)**. b)** Quantitation of (a) showing the percentage of RAD51 positive cells (error bars represent standard deviations). Experiment was done three times. c) DR-GFP assay was done to assess the HR proficiency in WT and *Rif1*$^{-/-}$ MEFs. DR-GFP reporter and pcBASceI constructs were co-transfected into WT and *Rif1*$^{-/-}$ MEFs. GFP positive cells were measured by flowcytometry, 48h post-transfection. 50,000 events were recorded for each sample. Percentage of GFP positive cells normalised to transfection efficiency of the respective cell line is plotted. The mean and SD from three independent experiments is represented, (ns, non-significant, Unpaired t-test). **d)** Representative images of SCE in WT and *Rif1*$^{-/-}$ MEFs with and without the treatment with HU and cisplatin. **e)** Quantitation of (d) showing the SCEs per cell (error bars represent standard deviations). 30 metaphases were analysed per sample and the experiment was repeated three times (ns, not significant, ****P<0.0001, Mann-Whitney test**). f)-g)** Western blot analysis for the downregulation of RAD51 in WT and RIF1$^{-/-}$ MEFs transfected with siControl or RAD51 smart pool 100 nmoles **(f)** and 50 nmoles **(g)**. Lysates prepared 48h post-transfection, were probed with antibody against RAD51 antibody. Tubulin is used as protein loading control. **h)** WT and *Rif1*$^{-/-}$ MEFs were transfected with siRad51 (50 nmols, 48h) followed by labeling and subsequent treatment with 4mM HU for 3h. IdU to CldU ratio was calculated to determine fork degradation in presence and absence of RAD51. Red bars represent mean values from a hundred and twenty five fibers from each genotype under each condition. The p-values were derived from Kruskal-Wallis ANOVA with Benjamini Hochberg (BH) post test. Experiment was repeated thrice with similar outcomes (Supplementary table 2 and Supplementary Figure.7f).

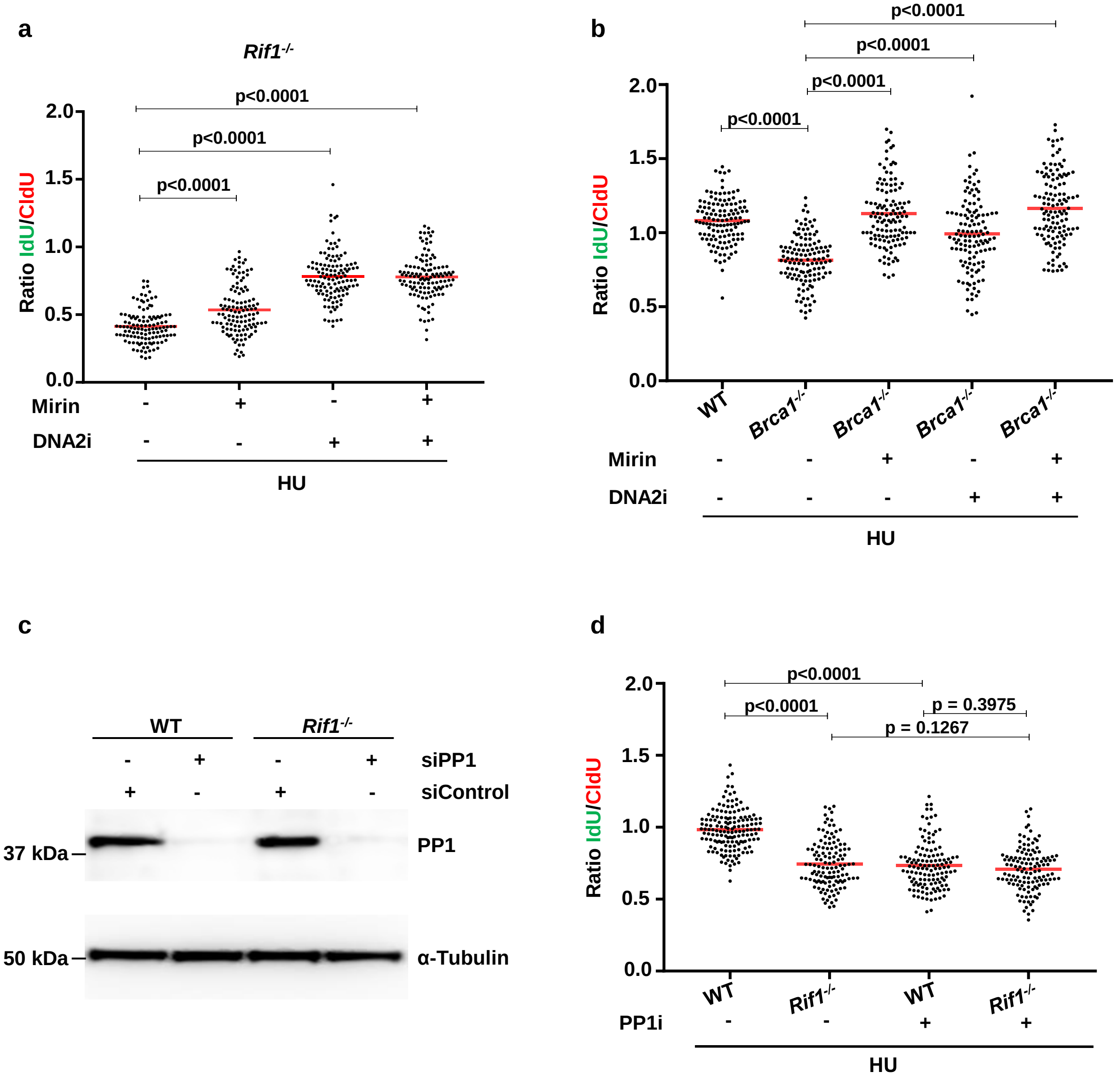

Supplementary Figure.4. Assessment of DNA2 mediated fork degradation in Rif1$^{-/-}$ MEFs using specific inhibitors for MRE11 and DNA2 and siPP1. a) DNA fibre analysis was done to determine the ratio of IdU/CldU in WT and *Rif1*$^{-/-}$ MEFs upon HU treatment after inhibiting MRE11 and DNA2 using mirin and NSC-105808 respectively. **b)** Ratio of IdU versus CldU upon HU treatment in WT and *Brca1*$^{-/-}$ with or without Mirin or DNA2i pre-treatment. **c)** Western blot analysis for the downregulation of PP1 in WT and RIF1$^{-/-}$MEFs. WT and RIF1$^{-/-}$ MEFs were transfected either with siControl or siPP1 smart pool. Lysates were prepared post 48h transfection and were probed with antibody against PP1 antibody. Tubulin is used as protein loading control. **d)** DNA fiber assay to assess fork degradation in WT and *Rif1*$^{-/-}$ MEFs pre-treated with 225nM tautomycetin (PP1i) for 2h and then subjected to treatment with 4mM HU for 3h. Red bars in **a, b** and **d** denotes mean. P-values for these experiments were obtained from Kruskal-Wallis ANOVA with Benjamini Hochberg post test. Experiment was repeated thrice with similar outcomes (Supplementary table 2, Supplementary Figure.7h,j and Supplementary Figure. 8c).

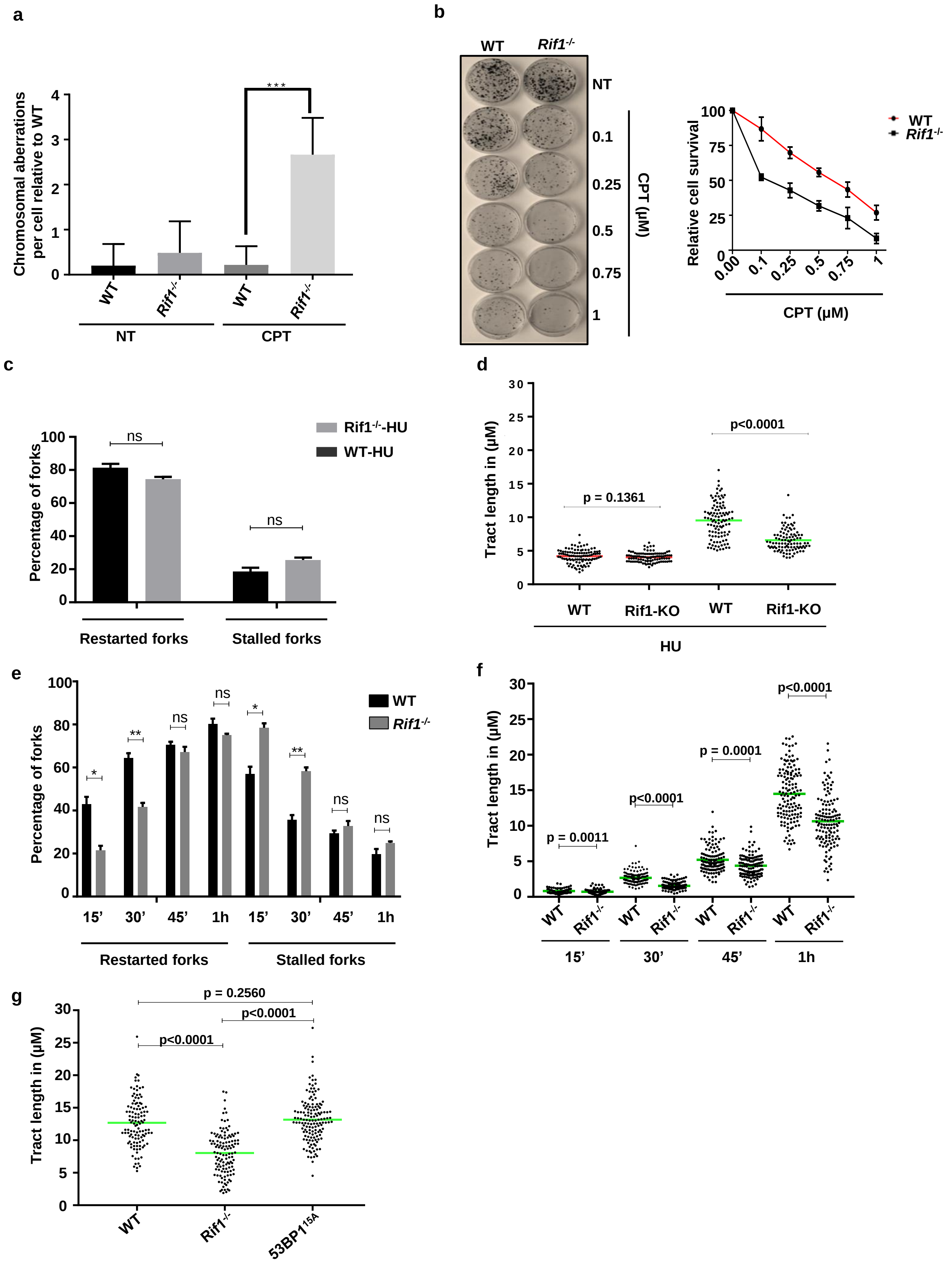
a
Chromosomal aberrations per cell relative to WT
4
3
2
1
0
***
WT
Rif1-/-
WT
Rif1-/-
NT
CPT
b
WT
Rif1-/-
NT
0.1
0.25
0.5
0.75
1
CPT (μM)
Relative cell survival
100
75
50
25
0
0.00
0.1
0.25
0.5
0.75
1
CPT (μM)
WT
Rif1-/-
c
Percentage of forks
100
80
60
40
20
0
ns
ns
Rif1-/--HU
WT-HU
Restarted forks
Stalled forks
d
Tract length in (μM)
30
25
20
15
10
5
0
p = 0.1361
p<0.0001
WT
Rif1-KO
WT
Rif1-KO
HU
e
Percentage of forks
100
80
60
40
20
0
*
**
ns
ns
*
**
ns
ns
WT
Rif1-/-
15'
30'
45'
1h
15'
30'
45'
1h
Restarted forks
Stalled forks
f
Tract length in (μM)
30
25
20
15
10
5
0
p = 0.0011
p<0.0001
p = 0.0001
p<0.0001
WT
Rif1-/-
WT
Rif1-/-
WT
Rif1-/-
WT
Rif1-/-
15'
30'
45'
1h
g
Tract length in (μM)
30
25
20
15
10
5
0
p = 0.2560
p<0.0001
p<0.0001
WT
Rif1-/-
53BP1 15A

Supplementary Figure.5. RIF1 deficiency leads to genome instability and delayed restart under replication stress. a) Genomic instability assessment after CPT treatment. Metaphase spreads were carried out in WT and *Rif1*$^{-/-}$ MEFs with and without CPT treatment and 60 metaphases were counted to calculate the chromosomal aberration per cell and plotted the graph (****$P \leq 0.0001$, Mann–Whitney test. **b)** Colony survival assay with and without CPT. WT and Rif1$^{-/-}$ MEFs were treated with varying concentrations of CPT for 4 hours and allowed to grow for 8 days. Adjoining graph is the quantitation of three independent experiments. **c)** Percentage of stalled and restarted forks in WT and *Rif1*$^{-/-}$ MEFs after treatment with HU followed by 1 h release. Graph represents the quantitation of three independent experiments (error bars represent standard deviations). **d)** Assessment of tract length in WT and RIF1-KO HAP1 cells after HU treatment. **e)** Percentage of restarted and stalled forks at different time points after HU treatment in WT and *Rif1*$^{-/-}$ MEFs. **f)** Assessment of IdU tract length in WT and *Rif1*$^{-/-}$ MEFs at different timepoints of restart after treatment with HU. **g)** Fork restart assay in WT, *Rif1* $^{-/-}$ and 53BP1^{15A} cells. All experiments were repeated thrice. Red bars in **d** (left panel) and green bars in **d** (left panel), **f** and **g** represents mean. P-value for **f** and **g** was derived from Kruskal-Wallis ANOVA with Benjamini Hochberg post test and from Mann Whitney test for **d**. Experiment was repeated thrice with similar outcomes (Supplementary table 2 and Supplementary Figure.8e,f).

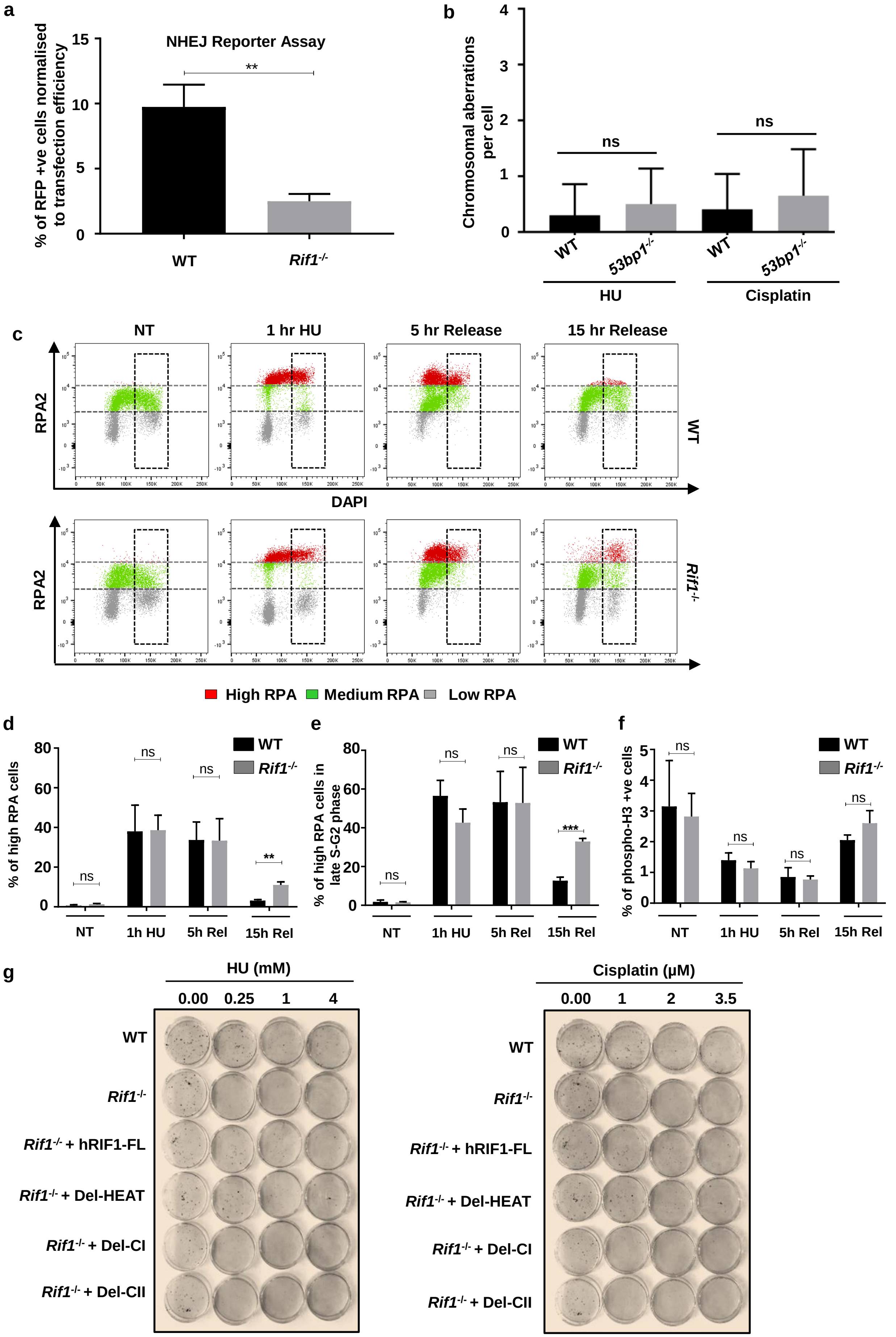
a
NHEJ Reporter Assay
% of RFP +ve cells normalised to transfection efficiency
**
0
5
10
15
WT
Rif1-/-
b
Chromosomal aberrations per cell
0
1
2
3
4
ns
ns
WT
53bp1-/-
WT
53bp1-/-
HU
Cisplatin
c
NT
1 hr HU
5 hr Release
15 hr Release
RPA2
DAPI
WT
Rif1-/-
High RPA
Medium RPA
Low RPA
d
% of high RPA cells
0
20
40
60
80
ns
ns
ns
**
WT
Rif1-/-
NT
1h HU
5h Rel
15h Rel
e
% of high RPA cells in late S-G2 phase
0
20
40
60
80
ns
ns
ns
***
WT
Rif1-/-
NT
1h HU
5h Rel
15h Rel
f
% of phospho-H3 +ve cells
0
1
2
3
4
5
ns
ns
ns
ns
WT
Rif1-/-
NT
1h HU
5h Rel
15h Rel
g
HU (mM)
0.00
0.25
1
4
Cisplatin (μM)
0.00
1
2
3.5
WT
Rif1-/-
Rif1-/- + hRIF1-FL
Rif1-/- + Del-HEAT
Rif1-/- + Del-CI
Rif1-/- + Del-CII

Supplementary Figure.6. **Delayed restart coupled to fork degradation in RIF1 deficient condition leads to accumulation of ssDNA resulting in genome instability . a)** Traffic Light assay was done to assess the NHEJ proficiency in WT and *Rif1*$^{-/-}$ MEFs by co-transfecting traffic light reporter and pcBASceI constructs and measuring RFP positive cells by flowcytometry. The mean and SD from three independent experiment is represented, (***P*= 0.0010, Unpaired t-test). **b)** Quantification of chromosomal aberrations in WT and *53bp*$^{-/-}$ MEFs upon HU and cisplatin treatment. Chromosomal aberrations in 60 metaphases per conditions was analyzed and three independent experiments and p value was calculated by unpaired t-test and presented in graph (ns non-significant). **c)** Measurement of ssDNA during cell cycle**.** WT and *Rif1*$^{-/-}$ MEFs were treated with and without HU treatment for 1h and samples were collected after the release time indicated on the top of each panel. Cells were counter stained with DAPI and RPA levels were determined by FACS. RPA level in WT and *Rif1*$^{-/-}$ MEFs at different time points is represented. Cells in late S-G2 phase is gated within the dotted box. Percentage of **d)** total high RPA positive cells, **e)** high RPA positive cells within late S-G2 phase population and **f)** phospho-histone3 positive cells under different condition is graphically represent. Data represents mean and SD from three independent experiments (ns, non significant, ***P*= 0.0019, ****P*= 0.0001; Unpaired t-test). **g)** hRIF1-FL and Del-HEAT complementation rescues the cell survival. Rif1$^{-/-}$ MEF cells were transfected with hRIF1-FL, Del-HEAT, Del-CI, and Del-C2 for 48h. Transfected cells were plated out at low dilutions and treated with HU and Cisplatin with different concentrations, and treatment was continued for 4h. After removal of the drug treated medium, cells were washed and allowed to grow in complete growth medium for 8 days. The colonies formed were stained and counted with cell counter. WT cells were taken as a control.

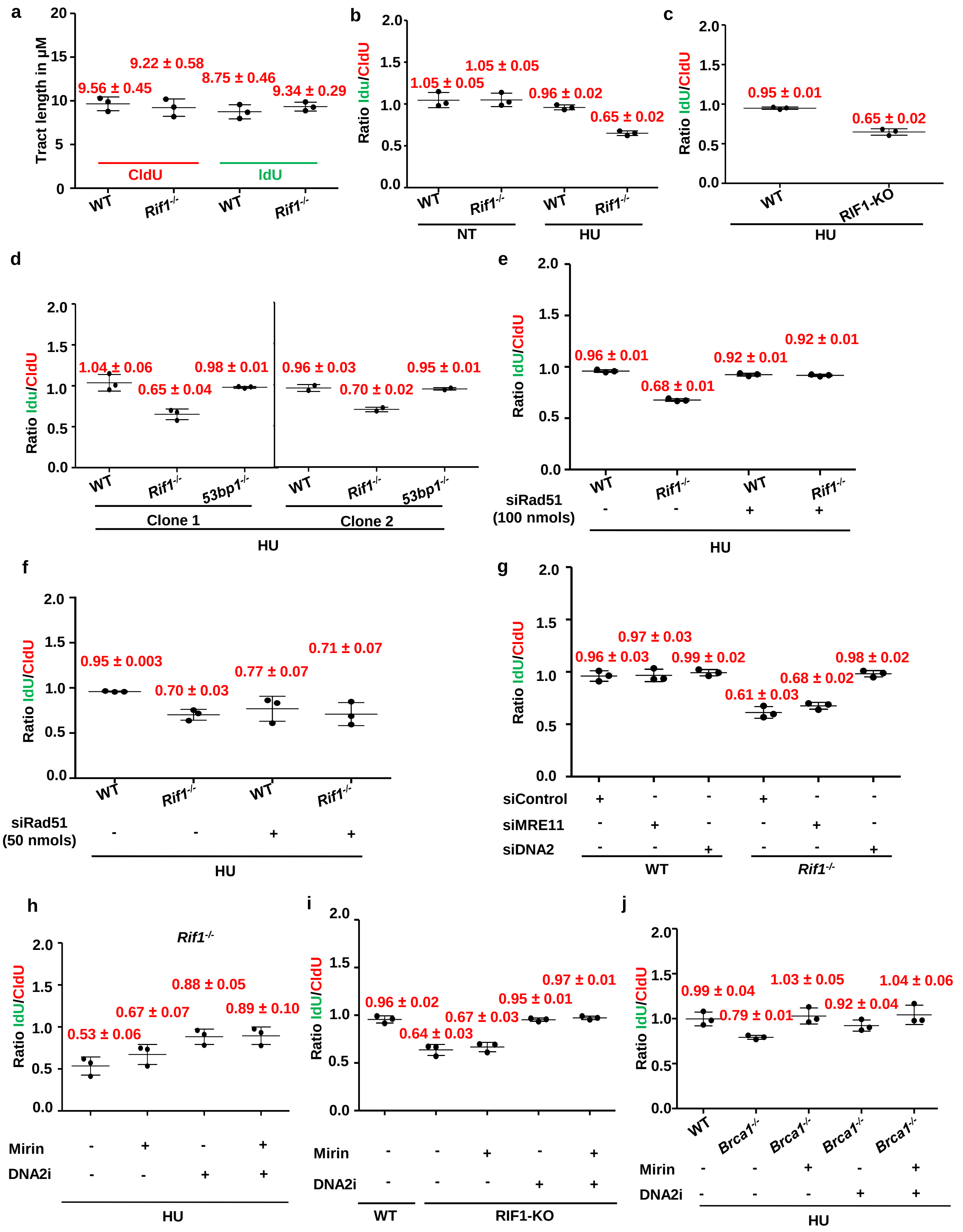

a
Tract length in μM
9.56 ± 0.45
9.22 ± 0.58
8.75 ± 0.46
9.34 ± 0.29
CldU
IdU
WT
Rif1-/-
WT
Rif1-/-
b
Ratio Idu/CldU
1.05 ± 0.05
1.05 ± 0.05
0.96 ± 0.02
0.65 ± 0.02
WT
Rif1-/-
WT
Rif1-/-
NT
HU
c
Ratio IdU/CldU
0.95 ± 0.01
0.65 ± 0.02
WT
RIF1-KO
HU
d
Ratio Idu/CldU
1.04 ± 0.06
0.65 ± 0.04
0.98 ± 0.01
0.96 ± 0.03
0.70 ± 0.02
0.95 ± 0.01
WT
Rif1-/-
53bp1-/-
WT
Rif1-/-
53bp1-/-
Clone 1
Clone 2
HU
e
Ratio IdU/CldU
0.96 ± 0.01
0.68 ± 0.01
0.92 ± 0.01
0.92 ± 0.01
WT
Rif1-/-
WT
Rif1-/-
siRad51 (100 nmols)
HU
f
Ratio IdU/CldU
0.95 ± 0.003
0.70 ± 0.03
0.77 ± 0.07
0.71 ± 0.07
WT
Rif1-/-
WT
Rif1-/-
siRad51 (50 nmols)
HU
g
Ratio IdU/CldU
0.96 ± 0.03
0.97 ± 0.03
0.99 ± 0.02
0.61 ± 0.03
0.68 ± 0.02
0.98 ± 0.02
siControl
siMRE11
siDNA2
WT
Rif1-/-
h
Rif1-/-
Ratio IdU/CldU
0.53 ± 0.06
0.67 ± 0.07
0.88 ± 0.05
0.89 ± 0.10
Mirin
DNA2i
HU
i
Ratio IdU/CldU
0.96 ± 0.02
0.64 ± 0.03
0.67 ± 0.03
0.95 ± 0.01
0.97 ± 0.01
Mirin
DNA2i
WT
RIF1-KO
j
Ratio IdU/CldU
0.99 ± 0.04
0.79 ± 0.01
1.03 ± 0.05
0.92 ± 0.04
1.04 ± 0.06
WT
Brca1-/-
Brca1-/-
Brca1-/-
Brca1-/-
Mirin
DNA2i
HU

Supplementary Figure.7. Mean and standard error of mean summarized for DNA fiber assay experiments in Supplementary table.2. Representative mean from three independent fibre experiments has been depicted by dot plots. Numbers in red depicts the mean of means and the standard error of means.**(a)-(e)** mean from independent experiments similar to Main Figure 2(a),(b),(c),(d) and (g); **(f)** depicted mean from biological replicates of Supplementary Figure 3(h); **(g)** mean from repeated experiments for Main Figure 3(b); **(h)** depicted mean from biological replicates of Supplementary Figure 4(a); **(i)** mean of means for Main Figure 3(c); **(j)** mean from independent experiments similar to Supplementary Figure 4(b).

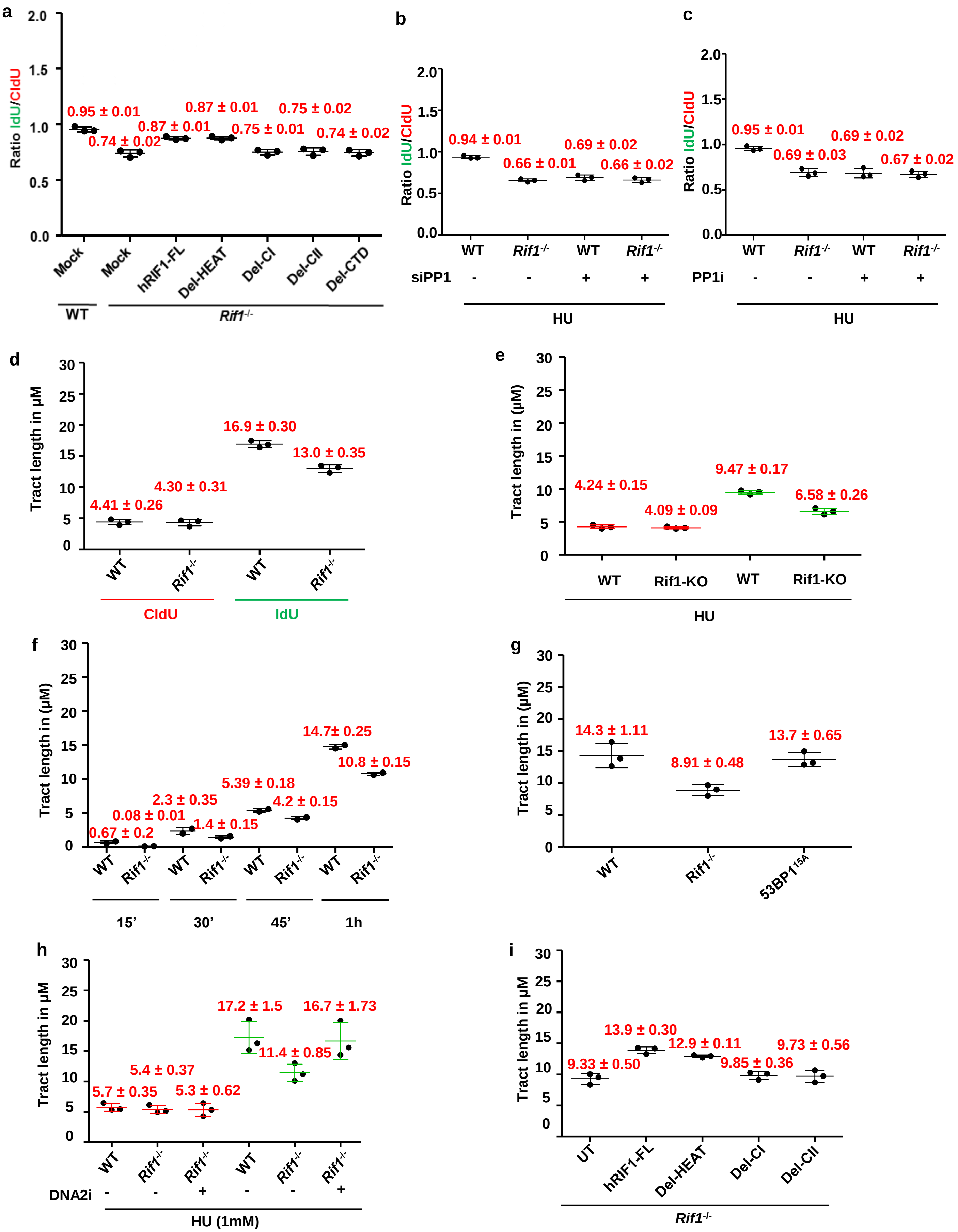
a
Ratio IdU/CldU
2.0
1.5
1.0
0.5
0.0
0.95 ± 0.01
0.74 ± 0.02
0.87 ± 0.01
0.87 ± 0.01
0.75 ± 0.01
0.75 ± 0.02
0.74 ± 0.02
Mock
Mock
hRIF1-FL
Del-HEAT
Del-CI
Del-CII
Del-CTD
WT
Rif1-/-
b
Ratio IdU/CldU
2.0
1.5
1.0
0.5
0.0
0.94 ± 0.01
0.66 ± 0.01
0.69 ± 0.02
0.66 ± 0.02
WT
Rif1-/-
WT
Rif1-/-
siPP1
-
-
+
+
HU
c
Ratio IdU/CldU
2.0
1.5
1.0
0.5
0.0
0.95 ± 0.01
0.69 ± 0.03
0.69 ± 0.02
0.67 ± 0.02
WT
Rif1-/-
WT
Rif1-/-
PP1i
-
-
+
+
HU
d
Tract length in µM
30
25
20
15
10
5
0
4.41 ± 0.26
4.30 ± 0.31
16.9 ± 0.30
13.0 ± 0.35
WT
Rif1-/-
WT
Rif1-/-
CldU
IdU
e
Tract length in (µM)
30
25
20
15
10
5
0
4.24 ± 0.15
4.09 ± 0.09
9.47 ± 0.17
6.58 ± 0.26
WT
Rif1-KO
WT
Rif1-KO
HU
f
Tract length in (µM)
30
25
20
15
10
5
0
0.67 ± 0.2
0.08 ± 0.01
2.3 ± 0.35
1.4 ± 0.15
5.39 ± 0.18
4.2 ± 0.15
14.7± 0.25
10.8 ± 0.15
WT
Rif1-/-
WT
Rif1-/-
WT
Rif1-/-
WT
Rif1-/-
15’
30’
45’
1h
g
Tract length in (µM)
30
25
20
15
10
5
0
14.3 ± 1.11
8.91 ± 0.48
13.7 ± 0.65
WT
Rif1-/-
53BP1 15A
h
Tract length in µM
30
25
20
15
10
5
0
5.7 ± 0.35
5.4 ± 0.37
5.3 ± 0.62
17.2 ± 1.5
11.4 ± 0.85
16.7 ± 1.73
WT
Rif1-/-
Rif1-/-
WT
Rif1-/-
Rif1-/-
DNA2i
-
-
+
-
-
+
HU (1mM)
i
Tract length in µM
30
25
20
15
10
5
0
9.33 ± 0.50
13.9 ± 0.30
12.9 ± 0.11
9.85 ± 0.36
9.73 ± 0.56
UT
hRIF1-FL
Del-HEAT
Del-CI
Del-CII
Rif1-/-

Supplementary Figure.8. Mean and standard error of mean summarized for DNA fiber assay experiments in Supplementary table.2 Representative mean from three independent fibre experiments has been depicted by dot plots. Numbers in red depicts the mean of means and the standard error of means. **(a)-(b)** mean from independent experiments similar to Main Figure 4(c) and (e); **(c)** depicted mean from biological replicates of Supplementary Figure 4(d); **(d)** mean from independent experiments similar to Main Figure 5(h); **(e)-(g)** depicted mean from biological replicates of Supplementary Figure 5(d),(f) and (g); **(h)-(i)** mean of means for Main Figure 6(g) and (h)**.**

Supplementary Table 1: List of Antibody used in this study.

Antibody	Catalogue Number, Source	Experiments	Dilutions
α-tubulin	ab56676, Abcam	Western blot	1:10000
Histone H3	ab1791, Abcam	Western blot	1:5000
XPD	ab150362, Abcam	Western blot	1:5000
PP1A	ab137512, Abcam	Western blot	1:2000
RPA32/2	ab2175, Abcam	Flowcytometry	1:200
phosphoH3	06-570, Merck Millipore	Flowcytometry	1:100
DNA2	PA568167, Invitrogen	Western blot, IP	1:1000
Phospho-(Ser/Thr)Phe	9631S, Cell Signalling technology	Western blot	1:1000
GFP	ab290, Abcam	Western blot	1:5000
BrdU (mouse)	347580, BD Biosciences	Fiber assay	1:100
BrdU (rat)	ab6326, Abcam	Fiber assay	1:100
hRif1	A300-568A, Bethyl	Western blot	1:1000
mRif1	gift from M. Di Virgilio, MDC	Western blot, IF, PLA	1:10000; 1:5000; 1:1000
Mre11	gift from A. Nussenzweig, NIH	Western blot	1:5000
53BP1	gift from R. Kanaar, Erasmus MC	Western blot, IF	1:1000
Rad51	gift from R. Kanaar, Erasmus MC	Western blot, IF	1:10000
Goat anti-hamster	PA1-29626, Thermo Fisher Scientific	Western blot	1:10000
Anti-Mouse IgG	NA931-1ML, GE Healthcare	Western blot	1:10000
Anti-Rabbit IgG	NA934-1ML, GE Healthcare	Western blot	1:10000

Supplementary Table 2: Fluorescent conjugated antibody used.

Antibody	Catalogue Number, Source	Experiments	Dilutions
anti-rat Cy3	712-166-153, Jackson Immuno Research	Fiber assay	1:250
anti-mouse IgG Alexa Fluor 488	A-11001, Invitrogen	Fiber Assay, IF	1:250, 1:1000
anti-mouse IgG Alexa Fluor 594	A-11005, Invitrogen	Flowcytometry; IF	1:150, 1:1000
anti-rabbit IgG Alexa Fluor 647	A-31573, Invitrogen	Flowcytometry	1:150
