# Supplementary material for: RIF1 promotes replication fork protection and efficient restart to maintain genome stability
Source: Nat Commun. 2019 Jul 23;10:3287. doi: 10.1038/s41467-019-11246-1 (PMC6650494; doi:10.1038/s41467-019-11246-1)
Supplement: Supplementary file 3 — Description of Additional Supplementary Files [file 41467_2019_11246_MOESM3_ESM.pdf]

Description of Additional Supplementary files

File name: Supplementary Data 1.

Description: IPOND data for identifying the proteins enriched and depleted at replication forks with and without replication stress with HU.

File name: Supplementary Data 2.

Description: Measurement of tract lengths and IdU/ CldU ratios from DNA Fiber Assay experiments.

File name: Supplementary Data 3.

Description: Electron microscopy data from all the experiments. Percentage of reversed forks (%RF) from 3 independent experiments for each sample is reported. Number of analyzed molecules is indicated within the parenthesis. **a)** Supplementary to Figure 2f. **b)** Supplementary to Figure 2h. **c)** Supplementary to Figure 3f. **d)** Supplementary to Figure 4d. **e)** Supplementary to Figure 4g.
