# Supplementary material for: RIF1 promotes replication fork protection and efficient restart to maintain genome stability
Source: Nat Commun. 2019 Jul 23;10:3287. doi: 10.1038/s41467-019-11246-1 (PMC6650494; doi:10.1038/s41467-019-11246-1)
Supplement: Supplementary file 6 — Supplementary Data 3 [file 41467_2019_11246_MOESM6_ESM.pdf]

a.

MEFs	WT		Rif1-/-	
HU (4mM)	-	+	-	+
%RF, Expt#1	5.5<br>(72)	49<br>(82)	5.6<br>(71)	21<br>(71)
%RF, Expt#2	7.0<br>(70)	42.8<br>(70)	5.7<br>(70)	24<br>(70)
%RF, Expt#3	7.0<br>(70)	44<br>(70)	8.0<br>(70)	24<br>(70)
**Mean, SD**	6.5, 0.866	45.33, 3.252	6.433, 1.358	22.93, 1.701

b.

MEFs	WT		Rif1-/-	
HU (4mM)	+	+	+	+
siRad51	-	+	-	+
%RF, Expt#1	41.0<br>(70)	5.7<br>(70)	25<br>(70)	4<br>(71)
%RF, Expt#2	39.0<br>(71)	8.5<br>(70)	25.2<br>(70)	6.49<br>(77)
%RF, Expt#3	40.28<br>(72)	8.97<br>(78)	24.6<br>(69)	6.58<br>(76)
**Mean, SD**	40.09, 1.013	7.723, 1.768	24.93, 0.3055	5.69, 1.464

c.

MEFs	WT		Rif1-/-	
HU (4mM)	+	+	+	+
Dna2i	-	+	-	+
%RF, Expt#1	49 (73)	48 (72)	25.7 (70)	47.8 (71)
%RF, Expt#2	41 (70)	50 (70)	25.7 (70)	42.8 (70)
%RF, Expt#3	41 (70)	47 (70)	22.8 (70)	44.3 (70)
**Mean, SD**	43.8, 4.508	48.33, 1.528	24.73, 1.674	44.97, 2.566

d.

MEFs	Rif1-/-				
HU (4mM)	+	+	+	+	+
Mutants	-	del C1	del C2	del heat	hRif FL
%RF, Expt#1	25.6 (74)	25.7 (70)	24.3 (70)	50 (70)	50 (70)
%RF, Expt#2	27.0 (70)	26.7 (70)	24.6 (70)	42.8 (70)	41 (70)
%RF, Expt#3	24.8 (70)	23.9 (70)	22.2 (70)	44.5 (70)	43 (70)
**Mean, SD**	25.8, 1.114	25.43, 1.419	23.7, 1.308	45.77, 3.763	44.67, 4.726

e.

MEFs	WT		Rif1-/-	
HU (4mM)	+	+	+	+
PP1i	-	+	-	+
%RF, Expt#1	40 (70)	21 (70)	23.6 (70)	27.5 (70)
%RF, Expt#2	41 (70)	29 (70)	27.0 (70)	27.5 (70)
%RF, Expt#3	43 (70)	27 (70)	23.5 (70)	28.5 (70)
**Mean, SD**	41.2, 1.311	24.7, 3.884	25.33, 1.992	27.67, 0.2887
