# Supplementary material for: RIF1 promotes replication fork protection and efficient restart to maintain genome stability
Source: Nat Commun. 2019 Jul 23;10:3287. doi: 10.1038/s41467-019-11246-1 (PMC6650494; doi:10.1038/s41467-019-11246-1)
Supplement: Supplementary file 7 — Reporting Summary [file 41467_2019_11246_MOESM7_ESM.pdf]

nature research

Corresponding author(s): NCOMMS-18-23072A

Last updated by author(s): 14/06/19

Reporting Summary

Nature Research wishes to improve the reproducibility of the work that we publish. This form provides structure for consistency and transparency in reporting. For further information on Nature Research policies, see Authors & Referees and the Editorial Policy Checklist.

Statistics

For all statistical analyses, confirm that the following items are present in the figure legend, table legend, main text, or Methods section.

n/a	Confirmed	
☐	☒	The exact sample size (*n*) for each experimental group/condition, given as a discrete number and unit of measurement
☐	☒	A statement on whether measurements were taken from distinct samples or whether the same sample was measured repeatedly
☐	☒	The statistical test(s) used AND whether they are one- or two-sided<br>*Only common tests should be described solely by name; describe more complex techniques in the Methods section.*
☒	☐	A description of all covariates tested
☐	☒	A description of any assumptions or corrections, such as tests of normality and adjustment for multiple comparisons
☐	☒	A full description of the statistical parameters including central tendency (e.g. means) or other basic estimates (e.g. regression coefficient) AND variation (e.g. standard deviation) or associated estimates of uncertainty (e.g. confidence intervals)
☐	☒	For null hypothesis testing, the test statistic (e.g. *F*, *t*, *r*) with confidence intervals, effect sizes, degrees of freedom and *P* value noted<br>*Give P values as exact values whenever suitable.*
☒	☐	For Bayesian analysis, information on the choice of priors and Markov chain Monte Carlo settings
☒	☐	For hierarchical and complex designs, identification of the appropriate level for tests and full reporting of outcomes
☒	☐	Estimates of effect sizes (e.g. Cohen's *d*, Pearson's *r*), indicating how they were calculated

Our web collection on statistics for biologists contains articles on many of the points above.

Software and code

Policy information about availability of computer code

Data collection	The following system was used for data collection:-<br>1. Wide-field fluorescent microscope: Carl Zeiss Axio Imager D2 microscope, 63X Plan Apo 1.4 NA oil immersion objective.<br>2. Confocal microscope: Zeiss LSM 700 Axio Imager Z2.<br>3. Electron microscope: FEI Talos, with 4K by 4K cmos camera.<br>4. Flow cytometry: BD LSRFortessa.<br>5. Gel imager: Uvidoc-HD2 gel imager.<br>6. Colony counter: Automated colony counter from Oxford Optronix Ltd.
Data analysis	The following softwares were used for data analysis:-<br>1. MaxQuant : for Mass-spectrometry data.<br>2. ImageJ : for measuring fibers and PFGE band intensities.<br>3. FlowJo v10.5.0 (BD Biosciences) : for flow cytometry data.<br>4. Adobe Photoshop CS4 : for making images.<br>5. Powerpoint : for making figures.<br>6. Excel : for recording readings and final datas.

For manuscripts utilizing custom algorithms or software that are central to the research but not yet described in published literature, software must be made available to editors/reviewers. We strongly encourage code deposition in a community repository (e.g. GitHub). See the Nature Research guidelines for submitting code & software for further information.

Data

Policy information about availability of data

All manuscripts must include a data availability statement. This statement should provide the following information, where applicable:

- Accession codes, unique identifiers, or web links for publicly available datasets
- A list of figures that have associated raw data
- A description of any restrictions on data availability

The iPOND dataset has been deposited in Figshare repository [https://doi.org/10.6084/m9.figshare.8242088.v1].
The raw datas for western blots, PFGE gels and Flowcytometry gating strategies are included within the Source Data file as separate sheets.

Field-specific reporting

Please select the one below that is the best fit for your research. If you are not sure, read the appropriate sections before making your selection.

☒ Life sciences ☐ Behavioural & social sciences ☐ Ecological, evolutionary & environmental sciences

For a reference copy of the document with all sections, see nature.com/documents/nr-reporting-summary-flat.pdf

Life sciences study design

All studies must disclose on these points even when the disclosure is negative.

Sample size	Sample sizes with respect to number of readings taken or the number of independent experiments are indicated in the respective figure legends.
Data exclusions	No data were excluded from the analysis.
Replication	Experiments were successfully replicated. Detailed readings from independent experiments are reported as supplementary information in form of tables.
Randomization	n/a
Blinding	Investigators were not blinded.

Reporting for specific materials, systems and methods

We require information from authors about some types of materials, experimental systems and methods used in many studies. Here, indicate whether each material, system or method listed is relevant to your study. If you are not sure if a list item applies to your research, read the appropriate section before selecting a response.

Materials & experimental systems

n/a	Involved in the study	
☐	☒	Antibodies
☐	☒	Eukaryotic cell lines
☒	☐	Palaeontology
☒	☐	Animals and other organisms
☒	☐	Human research participants
☒	☐	Clinical data

Methods

n/a	Involved in the study	
☒	☐	ChIP-seq
☐	☒	Flow cytometry
☒	☐	MRI-based neuroimaging

Antibodies

Antibodies used

Primary antibody used are:
mouse anti-tubulin (ab56676, Abcam); rabbit anti-histone H3 (ab1791, Abcam); rabbit anti-XPD (ab150362, Abcam); rabbit anti-PP1A (ab137512, Abcam); mouse anti-RPA32/2 (ab2175, Abcam); rabbit anti-phosphoH3 (06-570, Merck Millipore); rabbit anti-DNA2 (PA568167, Invitrogen); rabbit anti-Phospho-(Ser/Thr)Phe (9631S, Cell Signalling technology); rabbit anti-GFP(ab290, Abcam); mouse anti-BrdU(347580, BD Biosciences); rat anti-BrdU(ab6326, Abcam);rabbit anti-hRif1 (A300-568A, Bethyl); rabbit anti-mRif1 (gift from M. Di Virgilio, MDC); hamster anti-Mre11 (gift from A. Nussenzweig, NIH); mouse anti-53BP1 and rabbit anti-Rad51 (gift from R. Kanaar, Erasmus MC).
Secondary antibodies used are:
goat anti-hamster(PA1-29626, Thermo Fisher Scientific); ECL anti-Mouse IgG, HRP-linked whole Ab from sheep (NA931-1ML, GE Healthcare); ECL Rabbit IgG, HRP-linked whole Ab from donkey (NA934-1ML, GE Healthcare).
Fluorescent conjugated secondary antibodies used are:

	donkey anti-rat Cy3 (712-166-153, Jackson Immuno Research); goat anti-mouse IgG Alexa Fluor 488 (A-11001, Invitrogen); goat anti-mouse IgG Alexa Fluor 594 (A-11005, Invitrogen); donkey anti-rabbit IgG Alexa Fluor 647 (A-31573, Invitrogen).
Validation	All the antibodies used in the manuscript showed bands of expected size.

Eukaryotic cell lines

Policy information about cell lines

Cell line source(s)	1. WT MEF, Rif1-/- MEF, Brca1-/- MEF, 53BP1-/- MEF, 53BP1-15A MEF were from the Nussenzweig lab, NIH.<br>2. WT MEF (clone2), Rif1-/- MEF (clone2), 53BP1-/- MEF (clone2) were from Chapman lab, Welcome Trust Center For Human Genetics, University of Oxford.<br>3. WT HAP1, Rif1-/- HAP1 were from van Vugt Lab, University Medical Centre, Groningen.
Authentication	All the cell lines were authenticated by western blot using specific antibody against the genes which were knocked out.
Mycoplasma contamination	Cell lines were tested for Mycoplasma contamination.
Commonly misidentified lines<br>(See ICLAC register)	n/a

Flow Cytometry

Plots

Confirm that:

☒ The axis labels state the marker and fluorochrome used (e.g. CD4-FITC).

☒ The axis scales are clearly visible. Include numbers along axes only for bottom left plot of group (a 'group' is an analysis of identical markers).

☒ All plots are contour plots with outliers or pseudocolor plots.

☒ A numerical value for number of cells or percentage (with statistics) is provided.

Methodology

Sample preparation	Experimental procedures are provided in the Methods section.
Instrument	BD LSRFortessa was used for flow cytometry.
Software	FlowJo v10.5.0 (BD Biosciences)
Cell population abundance	n/a
Gating strategy	SSC-A/FSC-A (y/x) plots were used to remove cell debris and gate the population to be analyzed. Doublet elimination was done where ever required by plotting SSC-H against SSC-A.<br>1. Cell cycle analyses: EdU-A vs DAPI-A (y/x) were plotted.<br>2. RPA levels and phospho-H3 analysis: RPA-A vs DAPI-A (y/x) were plotted. Gating for high and low RPA population was done using unstained and RPA positive cells as controls. Increase or decrease in RPA level over the recovery time was relative to the respective non-treated samples. For detecting phospho-H3 positive cells and to map their position along with RPA levels, the population of phospho-H3 positive cells from the same sample were superimposed on the RPA-A vs DAPI-A (y/x) plot.<br>3. Plasmid based reporter assays:GFP- positive cells were gated by plotting GFP-A vs M-Cherry-A(y/x), where M-Cherry was used for negative gating. Gate for GFP positive cells were set by using GFP negative (non-transfected) cells and GFP positive (eGFP transfected) cells. For NHEJ Assay, the gating procedure was similar but a positive NHEJ event was read as a red signal (m-cherry +e) from the cell.<br>The figures show the percentage of cells based on the above mentioned gating strategy in FlowJo v10.5.0 software. Gating strategies are exemplified in source data.

☒ Tick this box to confirm that a figure exemplifying the gating strategy is provided in the Supplementary Information.
